# Human sulfotransferase *SULT2B1* physiological role and the impact of genetic polymorphism on enzyme activity and pathological conditions

**DOI:** 10.3389/fgene.2024.1464243

**Published:** 2024-08-30

**Authors:** Fatemah A. Alherz

**Affiliations:** Department of Pharmaceutical Science, College of Pharmacy, Princess Nourah bint Abdulrahman University, Riyadh, Saudi Arabia

**Keywords:** cholesterol sulfotransferase, *SULT2B1*, autosomal recessive ichthyosis, polymorphism, pregnenolone sulfotransferase, colon cancer

## Abstract

Human *SULT2B1*gene is responsible for expressing SULT2B1a and SULT2B1b enzymes, which are phase II metabolizing enzymes known as pregnenolone and cholesterol sulfotransferase (SULT), respectively. They are expressed in several tissues and contribute to steroids and hydroxysteroids homeostasis. Genetic variation of the *SULT2B1* is reported to be associated with various pathological conditions, including autosomal recessive ichthyosis, cardiovascular disease, and different types of cancers. Understanding the pathological impact of *SULT2B1* genetic polymorphisms in the human body is crucial to incorporating these findings in evaluating clinical conditions or improving therapeutic efficacy. Therefore, this paper summarized the most relevant reported studies concerning *SULT2B1* expression, tissue distribution, substrates, and reported genetic polymorphisms and their mechanisms in enzyme activity and pathological conditions.

## 1 Introduction

Human cytosolic Sulfotransferases (SULTs) are a family of phase II metabolizing enzymes that play a significant role in regulating several endogenous pathways involving synthesis, inactivation, and excretion of various endogenous compounds, including catecholamines, estrogen, thyroid hormones, bile acids, and hydroxysteroids (Liu and Klaassen, 1996; Falany, 1997; Strott, 2002). Cytosolic SULTs facilitate the transfer of the sulfonate group from a 3′-phosphoadenosine 5′-phosphosulfate (PAPS) molecule to an acceptor molecule containing amine or hydroxy group (Falany and Rohn-Glowacki, 2013). Most resulting metabolites become biologically inactive and eliminated via biliary or urinary excretion (Glatt et al., 2001; Strott, 2002).

Human cytosolic SUTLs contain thirteen enzymes categorized into four gene families named SULT1, SULT2, SULT4, and SULT6 (Allali-Hassani et al., 2007; Frank et al., 2011). Enzymes of the SULT2 family have been reported to exhibit the highest affinity in sulfating steroids and hydroxysteroid substrates (Glatt et al., 2001). In the SULT2 family, three isoforms have been identified: SULT2A1, SULT2B1a, and SULT2B1b, each exhibiting extinct affinity to a specific substrate and expressed in particular tissues, which influence their physiological role and involvement in different diseases (Her et al., 1996; Falany J. L. et al., 2006; Riches et al., 2009). SULT2A1, for instance, has a higher affinity toward dehydroepiandrosterone (DHEA), while SULT2B1a and SULT2B1b toward pregnenolone and cholesterol, respectively (Javitt et al., 2001; Falany and Rohn-Glowacki, 2013).

The role of SULT2B1 enzymes in sulfating endogenous substrates, especially steroids and hydroxysteroids, and their expression in hormonal-responsive tissues influence their roles in regulating the homeostasis of sex hormones (Higashi et al., 2004; Yanai et al., 2004; Falany C. N. et al., 2006; Koizumi et al., 2010; Salman et al., 2011; Yang et al., 2019). Therefore, interindividual variation in the *SULT2B1* gene may also impact physiological functions and pathological conditions. Thus, this paper aims to comprehensively review recently reported findings related to *SULT2B1* gene expression, tissue distribution, substrate specificity, and the impact of genetic polymorphism in enzyme activity and expression level and their role in pathological conditions.

## 2 Expression of SULT2b1 isoforms and tissue distribution

Human SULT2B1 cDNA was first cloned and characterized from the placenta, and its localization is mapped to the human chromosome 19 (Her et al., 1998). *SULT2B1* gene expression generates two mRNA isoforms, *SULT2B1a* and *SULT2B1b*, because of the different first exon used (Ji et al., 2007; Falany and Rohn-Glowacki, 2013). Therefore, the expressed isoenzymes (SULT2B1a and SULT2B1b) differ in the N-terminus region, and that influences their substrate specificity toward pregnenolone and cholesterol, respectively (Fuda et al., 2002; Ji et al., 2007; Falany and Rohn-Glowacki, 2013). The 25-residue of the N-terminus region of SULT2B1b was also identified as a part of allosteric binding site in *in silico* screening and molecular dynamic studies (Cook and Leyh, 2022). Moreover, compared to all reported SULT enzymes, SULT2B1a and SULT2B1b have unique extended carboxyl terminals rich with proline and serine residues, which were reported to be involved in enzymatic activity, thermostability, and subcellular localization (Lee et al., 2003; He and Falany, 2006).

Human *SULT2B1a* and *SULT2B1b* mRNA expression have been detected in several tissues like the prostate, placenta, small intestine, colon, trachea, lung, brain, and skin (Javitt et al., 2001). However, *SULT2B1b* mRNA is reported to be expressed several folds greater than *SULT2B1a* and in widespread tissues (Geese and Raftogianis, 2001; Javitt et al., 2001; Higashi et al., 2004; Yanai et al., 2004). Furthermore, only SULT2B1b protein has been detected in immunoblot analysis of human tissues and cell lines such as platelet, lung, skin, placenta, endometrium, and prostate (He et al., 2004; Higashi et al., 2004; Yanai et al., 2004; He et al., 2005; Koizumi et al., 2010; Zimmer et al., 2018). Besides normal tissues, SULT2B1b protein has also been detected in human cancerous tissues and cell lines involving prostate adenocarcinoma (LNCaP) and breast cancer cell lines (MCF-7, T47D) (Falany C. N. et al., 2006; He and Falany, 2007). Thus, most reported literature focuses on SULT2B1b enzyme.

## 3 SULT2B1 substrate specificity

As mentioned earlier, SULT2B1 enzymes exhibit activity toward hydroxysteroids, including cholesterol, pregnenolone, and DHEA (Javitt et al., 2001). Cholesterol plays crucial roles in the body, such as being the precursor for all steroid hormones, facilitating nutrient transportation, and activating cellular processes (Schade et al., 2020). SULT2B1b converts cholesterol into cholesterol sulfate, which is biologically active by itself (Javitt et al., 2001; Park J. H. et al., 2023). Cholesterol is also involved in the synthesis of pregnenolone, an initial precursor for the synthesis of steroid hormones, including DHEA (Falany and Rohn-Glowacki, 2013). Even though pregnenolone and DHEA are metabolized by several SULTs, with SULT2A1 exhibiting the highest activity, especially toward DHEA, SULT2B1b is reported to play a vital role in sulfating pregnenolone and DHEA due to its selective expression in important tissues like lung, skin, and reproductive tissues where SULT2A1 expression is low or not detectable (He et al., 2004; He et al., 2005). Therefore, it has been suggested that SULT2B1b could be involved in regulating DHEA, which is an essential precursor for reproductive hormones (Falany and Rohn-Glowacki, 2013). Furthermore, DHEA and pregnenolone, as well as their sulfated metabolites, act as neuromodulators, which are produced and released in the nervous system (brain) where SULT2B1b is expressed in higher levels (Vallee et al., 2001; Sanchez-Guijo et al., 2016; Vallee, 2016; Raciti et al., 2023).

SULT2B1b has also been reported to play an essential role in sulfating and regulating cholesterol-oxygenated derivatives (oxysterols), including 5α,6α-epoxycholesterol (5α,6α-EC), 5β,6β-epoxycholesterol (5 β,6 β -EC), 7-ketocholesterol (KC), 24-hydroxycholesterol (24HC), and 25-hydroxycholesterol (25HC) (Fuda et al., 2007; Cook et al., 2009; Ren and Ning, 2014). Oxysterols mediate several biological activities like sterol biosynthesis and act as signaling elements in several cellular processes (Vaya et al., 2011; Luu et al., 2016). Oxysterol sulfation by SUL2B1b may influence their biological activities and elimination (Fuda et al., 2007; Cook et al., 2009). For instance, SULT2B1b is reported to be able to convert KC, which is involved in atherosclerosis and retinal macular degeneration, into a sulfated metabolite, which prevents its cytotoxic effect (Fuda et al., 2007). Another study has also proven that inducing SULT2B1b expression in 293T cells mitigates the cytotoxic effect of KC by generating sulfated metabolite (Fuda et al., 2007; Bai et al., 2011). Furthermore, SULT2B1b was proposed to influence intracellular lipid homeostasis by catalyzing 25HC to its sulfated derivative (25HC-S), which is a main regulator of serum and hepatic lipid metabolism through its effects on the nuclear liver oxysterol receptor (LXR) and sterol regulatory element-binding proteins (SREBPs) (Bai et al., 2011; Bai et al., 2012).

In addition to endogenous substrates, SUlT2B1 enzymes were reported to exhibit minimal sulfating activity toward exogenous substrates and xenobiotics, like raloxifene, 4-n-nonylphenol, 3-OH-tibolone, p-nitrophenol, bisphenol A, 4-n-octylphenol, diethylstilbestrol, and 17-α-ethynylestradiol (Pai et al., 2002; Falany et al., 2004; Falany J. L. et al., 2006). SULT2B1b sulfating activity was also reported to be inhibited by endogenous cholesterol metabolite 7-dehydrocholesterol and antiandrogens like cyproterone, abiraterone, galeterone, and danazol (Yip et al., 2018; Cook and Leyh, 2022).

## 4 Effect of *SULT2B1* genetic variation and enzyme activity

According to the NCBI dbSNP database, a total of 19,975 single-nucleotide polymorphisms (SNPs) have been reported in the human *SULT2B1* gene, including 540 missense variants (NCBI, n.d.). Several studies have been conducted to study the effect of genetic variations, especially missense variants since they could lead to changes in the expressed amino acid and influence the function of the expressed enzymes. The first study was conducted on African American and Caucasian subjects and showed that *SULT2B1* genetic variations affect the expressed enzyme activities (Ji et al., 2007). The reported variants were eight coding missense variants in both SULT2B1b (Leu51Ser, Asp191Asn, Arg230His, and Peo345Leu) and SULT2B1a (Leu36Ser, Asp176Asn, Arg215His, and Pro330Leu) enzymes were expressed in COS-1 cells, and their activities were evaluated using DHEA as a substrate (Ji et al., 2007). The study showed that compared to the wild-type SULT2B1b and SULT2B1a, the tested allozymes displayed significant differential sulfating activities ranging from 76%–98% and 64%–88%, respectively (Ji et al., 2007). Another study examining the effect of ten missense *SULT2B1b* coding SNPs using cholesterol as a substrate showed a complete loss of the enzyme activity in three SULT2B1b allozymes (Gly72Val, Arg147His, and Gly276Val), while the rest of the allozymes (Pro69Ala, Thr73Met, Asp191Asn, Arg230His, Ser244Thr, Arg274Gln, and Pro345Leu) showed significant differential activities toward cholesterol between 0.5% and 54.6% of the wildtype activity (Table 1) (Alherz et al., 2018). Similar results were also reported for the same ten allozymes when their activities were evaluated using other endogenous substrates like DHEA and pregnenolone as substrates (Alherz et al., 2019). Furthermore, a study of 12 coding missense variations of SULT2B1a enzyme using pregnenolone as a substrate has reported no detectable activity of SULT2B1a-Arg132His, while the rest of the tested allozymes showed significant differential sulfating activity 0.5%–103.4% of the wildtype activity (Alatwi, 2022).

**TABLE 1:** Effect of single nucleotide polymorphisms on the enzymatic activity of SULT2B1a and SULT2B1b allozymes.

SNP ID	Amino acid substitution	Relative activity to wildtype (%)	Reference
SULT2B1b*			
rs777924668	Pro69Ala	4.5	Alherz et al. (2018)
rs746398875	Gly72Val	ND	
rs527454384	Thr73Met	3.5	
rs777140014	Arg147His	ND	
rs16982158	Asp191Asn	54.6	
rs16982169	Arg230His	49.1	
rs765224593	Ser244Thr	28.9	
rs762765702	Arg274Gln	0.5	
rs774212320	Gly276Val	ND	
rs17842463	Pro345Leu	37.2	
SULT2B1a**			
N.A.	Asp46Asn	1.7	Alatwi (2022)
rs777924668	Pro54Ala	16.0	
rs746398875	Gly57Val	4.4	
rs527454384	Thr58Met	1.0	
rs777140014	Arg132His	ND	
rs1114167424	Pro134Leu	89	
rs16982158	Asp176Asn	72	
rs16982169	Arg215His	103.4	
rs765224593	Ser229Thr	95.6	
rs762765702	Arg259Gln	1.1	
rs774212320	Gly261Val	0.5	
N.A.	Gly261Trp	1.2	
rs17842463	Pro330Leu	69.7	

*The enzyme activity of SULT2B1b was evaluated using 50 µM cholesterol.

**The enzyme activity of SULT2B1a was evaluated using 2.5 µM pregnenolone.

## 5 SULT2B1 genetic variations and diseases

As mentioned earlier, SULT2B1 is involved in metabolizing endogenous substrates such as steroids and hydroxysteroids and is highly expressed in hormone-responsive tissues, which suggests its critical role in physiological and pathophysiological conditions (Geese and Raftogianis, 2001; Higashi et al., 2004; Yanai et al., 2004; He et al., 2005; Koizumi et al., 2010; Salman et al., 2011). For instance, numerous studies reported the association of genetic variation or change in the expression of *SULT2B1* in various diseases and cancer types (Table 2) (He and Falany, 2007; Seo et al., 2013; Yang et al., 2013; Hu et al., 2015; Chen et al., 2016; Heinz et al., 2017; Youssefian et al., 2019; Fozia et al., 2021).

**TABLE 2:** Reported human SULT2B1 functional variants.

SNP type	Position^a^\Amino acid change SULT2B1a\SULT2B1b	SNP ID	Allele frequency	Disease	Reference
Intron	c.71 + 216T > C	rs279447	0.1254	Increase Endometrial cancer suitability	Low et al. (2010)
cSNP (synonymous)	c.789C > T (p.Cys263Cys)\ c.834C > T (p.Cys278Cys)	rs1132054	0.534447	Increase breast cancer suitability	Low et al. (2010)
Intron	c.782–436C > A\ c.827–436C > A	rs4149455	-	Reduced risk of esophageal squamous cell carcinoma	Hyland et al. (2013)
cSNP (synonymous)	c.903C > T (p.Asp301Asp)\ c.948C > T (p.Asp316Asp)	rs1052131	0.123736		
Intron	c.601–1246A > G\ c.646–1246A > G	rs12460535	0.645254	Correlated with prostate cancer progression and overall survival rate	Levesque et al. (2014)
Intron	c.170–2819T > A\ c.215–2819T > A	rs2665582	-		
Intron	c.378 + 1736A > G\c.423 + 1736A > G	rs10426628	0.761530		
Intron	c.72–1531C > A	rs3760808	-	Increase risk of prostate cancer	Koutros et al. (2011)
Intron	c.169 + 2144G > A\ c.214 + 2144G > A	rs10424237	-		
5′UTR	g.2878A > C	rs279451	0.191682	Linked with larger prostate volume	
cSNP (Missense)	c.401C > T (p.Pro134Leu)\ c.446C > T (p.Pro149Leu)	rs1114167424	-	Associated with autosomal-recessive congenital ichthyosis	Heinz et al. (2017)
cSNP (Missense)	c.776G > A (p.Arg259Gln)\ c.821G > A (p.Arg274Gln)	rs762765702	0.00002		
Inframe deletion	c.1054-1059delAGCCCC (p.Ser352-Pro353del)	rs16989366	0.00039		
cSNP (Missense)	c.187G > A (p.Glu63Lys)\ c.232G > A (p.Glu78Lys)	rs140526640	0.00008	Congenital ichthyosiform erythroderma	Youssefian et al. (2019)
cSNP (Missense)	c.253C > T (p.Arg85Trp)\ c.298C > T (p.Arg100Trp)	rs1303127476	-		
Intron	c.72–1967C > G	rs3760806	0.17872	Increased risk of colon cancer	Li et al. (2018)
Intron	c.169 + 4774C > G\ c.214 + 4774C > G	rs11878647	0.17173		

^a^ All referenced sequences are reported as cited in NCBI dbSNP; GRCh37.p13 chromosom 19, the accession numbers for SULT2B1a and SULT2B1b are NM_004605 and NM_177973, respectively. cSNP, coding single nucleotide polymorphism, 5′ UTR, 5′ untranslated region.

### 5.1 Skin-related disorder

Congenital ichthyosis is a skin disorder characterized by dry, scaling skin due to an imbalance in cholesterol sulfation and desulfation in the skin (Strott and Higashi, 2003). Four missense SNPs from exon coding SULT2B1b, p.Glu78Lys, p.Arg100Trp, p.Ala140Val, p.Arg274Gln, and p.Met304Ile were reported to be associated with the pathogenesis of autosomal recessive ichthyosis (Heinz et al., 2017; Youssefian et al., 2019; Fioretti et al., 2020; Fozia et al., 2021). One study reported that the change of glutamic acid to Lysine in SULT2B1bGlu78Lys caused a reduction in the cholesterol sulfating capacity of the enzyme, leading to the imbalance between cholesterol and cholesterol sulfate in the skin (Heinz et al., 2017). On the other hand, in SULT2B1bAla140Val, the change of alanine in location 140 into valine was proposed to disrupt the binding of SULT2B1b with the cofactor PAPS and affects the protein stability, which influences the activity of the enzyme in sulfating cholesterol efficiently (Fozia et al., 2021).

### 5.2 Cardiovascular disorder

A recent study has reported a possible link between the level of SULT2B1b in lymphocytes and acute myocardial infarction in patients with low levels of low-density lipoprotein (LDL) (Zhang Y. et al., 2020). The analysis of lymphocytes of those patients reveals a high expression level of SULT2B1b mRNA and protein, which is associated with high levels of cholesterol, inflammatory mediators like tumor necrosis factor-alpha (TNFα), and interferon-g (IFN- γ) in lymphocytes (Zhang Y. et al., 2020). Cholesterol accumulation in lymphocytes was reported to be promoted by SULT2B1b inhibition of LXR (Zhang Y. et al., 2020). LXR is activated by oxysterols such as 7 KC and 5 β,6 β -EC, and sulfation of oxysterols by SULT2B1b converts them into sulfated metabolites, which then act as antagonists of LXR (Song et al., 2001; Zhang Y. et al., 2020). Sulfated oxysterols may also play a role in atherosclerosis by promoting *de novo* cholesterol synthesis and apoptosis in several cell types, including macrophages (Song et al., 2001). Additionally, a recent study has reported that *SULT2B1* expression is elevated with the progression of atherosclerosis (Pan et al., 2024). In fact, the knockdown of *SULT2B1* in animal models was suggested to promote atherosclerosis remission and reduce inflammatory mediator levels (Pan et al., 2024). The study explains that lowering the expression of *SULT2B1* in macrophages leads to a reduction of 25HC-S production, which then increases the expression of LXR, suppressing the activation of macrophages via nuclear factor κB (NF-κB) and attenuating inflammation accordingly (Pan et al., 2024). It was also reported that *SULT2B1b* knockdown upregulates miR148a-3p and inhibits IκB kinase β (IKKβ)\ NF-κB signaling pathway in macrophage, which reduces inflammation (Yin et al., 2021; Zhang J. et al., 2023). On the other hand, overexpression of SULT2B1 in mice (under high cholesterol diet) inhibits LXR, which reduces the high-density lipoprotein (HDL) levels and, as a consequence, reduces the beneficial effects of HDL in lowering cholesterol levels through activation of reverse cholesterol transport (Nishida et al., 2024). Finally, genotyping of monocytes in coronary artery disease patients has shown that the *SULT2B1* genetic variant in the promoter region (rs2665580), especially with the GG genotype, is associated with a high expression level of *SULT2B1*, which corresponds with increased inflammatory factors and unstable coronary plaques (Pan et al., 2024).

### 5.3 Cancer-related to reproductive Organs

*SULT2B1* intron variants (rs12460535, rs2665582, and rs10426628) were found to be correlated with prostate cancer progression and overall survival rate (Levesque et al., 2014). The study suggested that *SULT2B1* (rs10426628) lowers the risk of prostate cancer progression by reducing the circulating steroid hormones via forming less active sulfated metabolites (Levesque et al., 2014). *In vivo* and *in vitro* studies demonstrated that inhibiting SULT2B1b enzyme expression in prostate cancer cells could promote prostate cancer proliferation in response to DHEA treatment (He and Falany, 2007; Seo et al., 2013). In fact, data from the Protein Atlas shows that the low expression level of *SULT2B1* is significantly (*p* = 0.044) and inversely correlated with the 5-year survival rate (Yang et al., 2019). These findings were confirmed in a clinical study using isolated human prostate cancer tissue samples, which reported that SULT2B1b enzyme is expressed at a very low level in advanced metastatic prostate cancer compared to normal prostate, suggesting SULT2B1b expression may offer a protective effect by reducing the availability of active steroid hormone precursors like DHEA (He and Falany, 2007; Seo et al., 2013). Furthermore, in castration-resistant prostate cancer, androgen biosynthesis from adrenal DHEA is mediated by the action of aldo-keto reductase (AKR)1C3, which promotes cancer growth and invasion by activating androgen receptors (Park et al., 2020). SULT2B1b depletion was reported to promote AKR1C3 expression, which activates extracellular-signal-regulated kinase 1/2 (ERK1/2) tumor cell survival signal, activates androgen receptors, and induces epithelial-to-mesenchymal (EMT)-like changes that promote cancer progression and invasiveness (Park et al., 2020). Interestingly, another study has reported contradictory results, demonstrating that *SULT2B1b* knockdown increases TNFα expression in prostate cancer, promoting TNF-mediated apoptosis (Vickman et al., 2019). Similarly, another *in vitro* study has shown that *SULT2B1b* knockdown reduces prostate cancer cell growth and viability and promotes cell death (Vickman et al., 2016).

On the other hand, in breast cancer, *SULT2B1* expression was reported to be upregulated in both estrogen receptor a (ERa) positive and negative breast cancer tissues, with a higher level in ER-positive tumors (Bieche et al., 2004; Tozlu et al., 2006; Low et al., 2010; Hevir et al., 2011). Similarly, *SULT2B1* is also reported to be expressed at a high level in endometrial cancer, cervical cancer, and ovarian cancer, which negatively impacts prognosis (Dumas et al., 2008; Low et al., 2010; Hevir et al., 2011; Zhang Y. et al., 2023; Gao et al., 2024). Downregulation of *SULT2B1* in ovarian cancer cell lines reduces cell proliferation, migration, and invasion by binding to annexin A9 (ANXA9) and regulates its expression (Gao et al., 2024). ANXA9 is a calcium-dependent phospholipid-binding protein that has been reported to promote different cancer development and chemotherapy resistance (Boudhraa et al., 2016; Kou et al., 2021; Zhang et al., 2021; Zhou et al., 2021). Furthermore, inhibiting the expression of *SULT2B1* with small molecules like verteporfin was reported to inhibit cervical cancer cell proliferation, migration, and invasion and promote cell apoptosis (Yin and Chen, 2020).

### 5.4 Gastrointestinal cancers

Genetic analysis of esophageal squamous cell carcinoma (ESCC) reveals that the *SULT2B1* rs4149455 intronic variant and rs1052131 synonymous variant correlate with reduced cancer risks (Hyland et al., 2013). A clinical study on patient samples of ESCC demonstrated that SULT2B1 expression level is reduced or even abolished in ESCC tissues compared to matched adjacent normal epithelial cells (Li et al., 2021). The study suggested that reduced expression of SULT2B1 upregulates Per1 gene expression, a circadian clock gene that is involved in tumor initiation and malignant progression (Li et al., 2021). Functional analysis proves that SULT2B1 overexpression *in vitro* reduces tumor cell proliferation and retard tumor growth *in vivo*, while SULT2B1b knockdown promotes ESCC progression (Yue et al., 2017; Li et al., 2021). Another study proposed different mechanisms, which suggest that SULT2B1enzymes promote the development of ESCC by sulfating DHEA, leading to a reduced pool of sex hormones such as estradiol (Hyland et al., 2013). Estradiol treatment of ESCC cell lines inhibited the viability and migration ability of cancerous cells (Wang et al., 2020). A clinical study has also shown that low estradiol levels increase the incidence of developing ESCC (Wang et al., 2011).

In normal gastric epithelial cells, SULT2B1 expression was reported to promote their repair after damage and play a protective role in preventing gastric carcinogenesis induced by 3-methylcholanthrene (a carcinogenic agent) (Hong et al., 2019). However, elevated expression of SULT2B1b mRNA and protein in gastric cancer tissue was reported to promote tumor angiogenesis, lower the survival rate, and affect prognosis negatively (Chen et al., 2016). The SULT2B1 expression level was also correlated with gastric cancer stage and proposed to be used as an independent biomarker for gastric cancer prognosis (Chen et al., 2016).

In colorectal cancer, SULT2B1b expression was reported to be upregulated, which could promote disease progression and lower disease-specific survival and disease-free rates; thus, it was proposed to be used as a prognostic biomarker and a potential therapeutic target (Hu et al., 2015; Zhang Z. Y. et al., 2020; Tatsuguchi et al., 2022b; Che et al., 2024). *In vitro* study SULT2B1b knockdown suppresses colorectal cancer cell growth and migration, suggesting the vital role of SULT2B1b in colorectal cancer cell proliferation and invasion (Hu et al., 2015; Che et al., 2024). The study also showed that SULT2B1 facilitates lipid metabolism and promotes colon cancer cell metastasis by interacting directly with stearoyl-CoA desaturase (SCD1), which is involved in lipid metabolism (Che et al., 2024). Furthermore, another study has reported that SULT2B1 is highly expressed in chemoresistance colon cancer and radio-resistance tissues, which promotes cell proliferation and chemoresistance in colon cancer through its involvement in oncogenic signaling involving the OLR1/c-MYC/SULT2B1 axis (Zhao et al., 2021; Huang et al., 2022). Oxidized low-density lipoprotein receptor 1 (OLR1) promotes SULT2B1 expression by increasing c-MYC expression while knockdown the ORL1 downregulating c-MYC expression, which results in reducing SULT2B1 level as well as reduced glycolytic metabolism leading to decreased cancer cell growth and chemoresistance in colon cancer (Zhao et al., 2021; Huang et al., 2022). Furthermore, SULT2B1b was proposed to increase cancer cell resistance to immunotherapy by promoting the production of cholesterol sulfate (Tatsuguchi et al., 2022b). For instance, T cells treated with cholesterol sulfate showed a reduction in the immune response by reducing T-cell receptor signaling through disturbing T-cell microvilli function (Park J. S. et al., 2023). Furthermore, cholesterol sulfate inhibits dedicator of cytokinesis protein 2 (DOCK2), a Rac activator crucial for lymphocyte activation and migration, decreasing CD8^+^ T cell infiltration into colon cancer tissues (Sakurai et al., 2018; Tatsuguchi et al., 2022b; Morino et al., 2023; Wang et al., 2023). *In vivo*, inhibition of SULT2B1b with 3b-hydroxy-5-cholenoic acid promotes CD8^+^ T cell infiltration to cholesterol-sulfate-producing tumor and sensitizes the tumor to immunotherapy (Tatsuguchi et al., 2022a).

Similarly, *in vitro* and *in vivo* studies in hepatocellular carcinoma showed that SULT2B1b is overexpressed in cancer tissues and associated with cell proliferation and migration (Yang et al., 2013; Wang et al., 2019). The knockdown of SULT2B1b *in vitro* suppresses tumor cell growth and promotes apoptosis and cell cycle arrest in Hepa1-6 cells by increasing the pro-apoptotic factor (FAS) expression, and downregulating the anti-apoptotic factor BCL-2, cyclinB1, and MYC, promoting cancer cell death (Yang et al., 2013). SULT2B1b overexpression may also promote tumor growth by indirectly inhibiting the LXR by sulfated oxysterols, which prevents the antiproliferative, anti-inflammatory, and lipid-regulating activities of LXR (Uppal et al., 2007; Zhang et al., 2012). Wang et al. have also reported that the SULT2B1-cholesterol sulfate-DOCK2 axis plays a vital role in inhibiting CD8^+^ T cell infiltration to the tumor microenvironment of hepatocellular carcinoma, which may affect immunotherapy efficacy (Wang et al., 2023). Thus, it was suggested to target the SULT2B1-cholesterol sulfate-DOCK2 axis to improve immunotherapy efficacy (Seimiya et al., 2023; Wang et al., 2023).

### 5.5 Urinary tract cancer

SULT2B1 was also reported to be expressed at a high level in kidney cancer compared to normal tissues, and that correlated with poor prognosis (Li et al., 2019). Overexpression of SULT2B1 promotes cancer cell proliferation by reducing tumor infiltration with lymphocytes as well as reducing the expression level of macrophages, neutrophils, B cells, CD4^+^ cells, CD8^+^ cells, and dendritic cells (Li et al., 2019). On the other hand, SULT2B1 knockdown inhibits cancer cell proliferation and reduces invasion and migration (Li et al., 2019).

## 6 Conclusion and future directions

SULT2B1b is a phase II metabolizing enzyme that plays a key role in regulating the homeostasis of several steroids, hydroxysteroids, and oxysterols in various body tissues, as well as molecular signaling pathways. SULT2B1 displays interindividual genetic variability in different ethnic groups (Glatt and Meinl, 2004). The altered activity or expression level of SULT2B1 due to coding or non-coding SNPs could have clinical consequences influencing disease susceptibility, drug response, and normal physiological function (Daniels and Kadlubar, 2013; Mueller et al., 2015). In fact, SULT2B1 genetic variation has been proven to be involved in various disease conditions like autosomal recessive ichthyosis, cardiovascular disease, and various malignancies. Animal studies, *in vitro* studies, and clinical data reported that aberrant expression of SULT2B1b has been associated with different cancer progression and tumor growth. An increased expression of SULT2B1 in colorectal, breast, endometrial, and liver cancers has been reported to promote tumor growth and poor prognosis. In contrast, reduced expression in ESCC and prostate cancer promotes tumor growth and negatively impacts survival rates (Hu et al., 2015). Furthermore, SULT2B1 expression level has been suggested to be used as an oncogenic marker for colon cancer stage and prognosis.

To date, studies conducted to study the association between *SULT2B1* genetic variation and disease susceptibility or drug response are limited or have contradictory results. Thus, more genome-wide association studies and functional studies on SULT2B1 will help predict individual suitability to different diseases and drug responses.
